# The Impact of Improved Water, Sanitation, and Hygiene on Oral Rotavirus Vaccine Immunogenicity in Zimbabwean Infants: Substudy of a Cluster-randomized Trial

**DOI:** 10.1093/cid/ciz140

**Published:** 2019-03-29

**Authors:** James A Church, Sandra Rukobo, Margaret Govha, Benjamin Lee, Marya P Carmolli, Bernard Chasekwa, Robert Ntozini, Kuda Mutasa, Monica M McNeal, Florence D Majo, Naume V Tavengwa, Lawrence H Moulton, Jean H Humphrey, Beth D Kirkpatrick, Andrew J Prendergast

**Affiliations:** 1 Zvitambo Institute for Maternal and Child Health Research, Harare, Zimbabwe; 2 Centre for Genomics and Child Health, Blizard Institute, Queen Mary University of London, United Kingdom; 3 Vaccine Testing Center, Larner College of Medicine, University of Vermont, Burlington; 4 Department of Pediatrics, Larner College of Medicine, University of Vermont, Burlington; 5 Department of Microbiology and Molecular Genetics, Larner College of Medicine, University of Vermont, Burlington; 6 Department of Pediatrics, University of Cincinnati College of Medicine, Division of Infectious Diseases, Cincinnati Children’s Hospital Medical Center, Ohio; 7 Department of International Health, Johns Hopkins Bloomberg School of Public Health, Baltimore, Maryland

**Keywords:** infants, Africa, rotavirus, oral vaccine, WASH

## Abstract

**Background:**

Oral vaccines have lower efficacy in developing compared to developed countries. Poor water, sanitation, and hygiene (WASH) may contribute to reduced oral vaccine immunogenicity.

**Methods:**

We conducted a cluster-randomized 2 × 2 factorial trial in rural Zimbabwe. Pregnant women and their infants were eligible if they lived in clusters randomized to (1) standard of care (52 clusters); (2) improved infant feeding (53 clusters); (3) WASH: ventilated improved pit latrine, 2 hand-washing stations, liquid soap, chlorine, infant play space, and hygiene counseling (53 clusters); or (4) feeding plus WASH (53 clusters). This substudy compared oral rotavirus vaccine (RVV) seroconversion (primary outcome), and seropositivity and geometric mean titer (GMT) (secondary outcomes), in WASH vs non-WASH infants by intention-to-treat analysis.

**Results:**

We included 801 infants with documented RVV receipt and postvaccine titer measurements (329 from 84 WASH clusters; 472 from 102 non-WASH clusters); 328 infants with prevaccination titers were included in the primary outcome. Thirty-three of 109 (30.3%) infants in the WASH group seroconverted following rotavirus vaccination, compared to 43 of 219 (19.6%) in the non-WASH group (absolute difference, 10.6% [95% confidence interval {CI}, .54%–20.7%]; *P* = .031). In the WASH vs non-WASH groups, 90 of 329 (27.4%) vs 107 of 472 (22.7%) were seropositive postvaccination (absolute difference, 4.7% [95% CI, –1.4% to 10.8%]; *P* = .130), and antirotavirus GMT was 18.4 (95% CI, 15.6–21.7) U/mL vs 14.9 (95% CI, 13.2–16.8) U/mL (*P* = .072).

**Conclusions:**

Improvements in household WASH led to modest but significant increases in seroconversion to RVV in rural Zimbabwean infants.

**Clinical Trials Registration:**

NCT01824940.

## Teaser

Oral vaccines have lower efficacy in developing compared to developed countries. In a substudy of a cluster-randomized trial, improvements in household water, sanitation, and hygiene led to modest increases in seroconversion to oral rotavirus vaccine among rural Zimbabwean infants.

Rotavirus diarrhea causes approximately 215 000 deaths annually, predominantly in sub-Saharan Africa and south Asia [1]. A key preventive strategy is oral rotavirus vaccination, which had been introduced in 96 countries by October 2018 [2]. However, oral vaccines consistently underperform in settings where most diarrheal deaths occur. For example, oral rotavirus vaccine (RVV) protective efficacy against severe rotavirus gastroenteritis in Europe is 98% [3], compared to 39% in Ghana, Kenya, and Mali [4], and seroconversion to RVV is consistently lower in developing compared with developed countries [5].

Multiple intestinal factors have been implicated in oral vaccine underperformance [6], including enteropathogens [7], diarrhea [8], and perturbations in the commensal microbiota [9], which may decrease the effective titer of vaccine virus. Additionally, environmental enteric dysfunction, a chronic condition of altered gut structure and function [10] might prevent vaccine uptake and attenuate the mucosal immune response to vaccine antigens [11, 12]. Fecal contamination due to poor water, sanitation, and hygiene (WASH) may contribute to this intestinal pathology and reduced oral vaccine immunogenicity.

Improving oral vaccine performance would reduce morbidity and mortality from enteric disease, but most interventions have shown no benefit [13]. No trials have evaluated the impact of WASH on oral vaccine performance. We hypothesized that a WASH intervention could enhance immune responses to RVV in early infancy. We conducted a nested substudy within the Sanitation Hygiene Infant Nutrition Efficacy (SHINE) trial to test this hypothesis.

## METHODS

### Study Population

SHINE was a 2 × 2 factorial, cluster-randomized trial across 2 districts in rural Zimbabwe, which tested the independent and combined effects of improved WASH and improved infant and young child feeding (IYCF) on child length-for-age and hemoglobin at 18 months of age. The trial design, procedures, and outcomes have been reported elsewhere [14, 15]. In brief, pregnant women were enrolled between November 2012 and March 2015 from clusters randomized to 1 of 4 arms: standard of care, IYCF, WASH, or combined IYCF and WASH. WASH households had a ventilated improved pit latrine [16] and 2 hand-washing stations [17] installed within 6 weeks of enrollment (approximately 20 weeks’ gestation), and received monthly liquid soap, water chlorination solution (WaterGuard, Nelspot, Zimbabwe), an infant play space, and mat. A subgroup of infants underwent longitudinal specimen collection at 1, 3, 6, 12, and 18 months of age [18].

### Rotavirus Substudy

The rotavirus substudy was a prespecified objective in the SHINE trial protocol; the analysis plan was published prior to unblinding (https://osf.io/ad9zr/). In May 2014, midway through the trial, oral rotavirus vaccine (Rotarix) was introduced in Zimbabwe and administered with oral polio vaccine at 6 and 10 weeks of age. Vaccination was undertaken at local clinics and not overseen by the trial; national rotavirus vaccination coverage in 2015–2016 was 87%–91% [19]. Dates of vaccine receipt were recorded through review of child health cards, with each child’s final vaccination status categorized as complete (2 doses), incomplete (1 dose), or no vaccine (zero doses).

Infants were eligible for the substudy if they were human immunodeficiency virus (HIV) unexposed, live-born after introduction of rotavirus vaccine, and had at least 1 stored plasma sample (minimum 100 uL) collected before 6 months of age. Available plasma for each infant was allocated to pre- and postvaccination time-points, based on the documented date of receipt of the first dose of RVV. Infants were excluded from analysis if they had no postvaccine titer measurement, had missing data on vaccination, or had not received at least 1 dose of rotavirus vaccine.

The primary outcome of this substudy was the proportion of infants with RVV seroconversion, defined as a postvaccine plasma concentration of antirotavirus immunoglobulin A (IgA) ≥20 U/mL in infants who were seronegative (<20 U/mL) prevaccination [20]. Secondary outcomes were the proportion of seropositive infants (defined as postvaccine titer ≥20 U/mL) and geometric mean titer (GMT). Antirotavirus IgA is the most widely used marker of vaccine response or natural infection [5]. We measured antirotavirus IgA titers in cryopreserved plasma using an enzyme-linked immunosorbent assay, as previously described [21]. The assay lower limit of detection was 7.5 U/mL. Laboratory assays and data analysis were conducted blind to trial arm.

Our sample size calculation was based on published seroconversion rates from African rotavirus vaccine trials of 47.2%–57.1% [22]. Assuming a coefficient of variation of 0.25, 360 infants per group would provide 80% power to detect a 15% increase in RVV seroconversion between WASH and non-WASH groups. These calculations assumed an arithmetic mean of 5 infants per cluster and a harmonic mean of 3 per cluster (to adjust for variability in cluster size).

**Ethical Considerations and Data Availability**

The original SHINE trial and this laboratory substudy were approved by the Medical Research Council of Zimbabwe and the John Hopkins School of Public Health Committee on Human Research. Written informed consent was obtained from all mothers prior to enrollment in the trial.

The full protocol and statistical analysis plan for the SHINE trial are available at https://osf.io/w93hy. Data collected for the SHINE trial will be made publicly available as individual participant data with an accompanying data dictionary. The data will be housed and made accessible to the global research community through Clinical Epidemiology Database Resources (ClinEpiDB) (http://ClinEpiDB.org) at the University of Pennsylvania. This platform is charged with ensuring that epidemiological studies are fully anonymized by removing all personal identifiers and obfuscating all dates per participant through application of a random number algorithm to comply with the ethical conduct of human subjects research. Researchers must agree to the policies and comply with the mechanism of ClinEpiDB to access data housed on this platform.

### Statistical Analysis

Analyses were intention-to-treat. We used generalized estimating equations accounting for within-cluster correlation, containing 2 dummy variables representing the WASH and IYCF interventions, unadjusted for other covariates, and with an exchangeable working correlation structure [23]. Because there was no statistical interaction between interventions, we compared all outcomes between the combined WASH and IYCF + WASH arms (“WASH group”) and the combined standard of care and IYCF arms (“non-WASH group”). We used a log-binomial specification to estimate relative risks (RRs) for dichotomous outcomes. For antirotavirus GMT, we used a log-normal censored regression model (Tobit), with left censoring at 7.5 U/mL. We used other methods to adjust for within-cluster correlation including multinomial and ordinal regression models with robust variance estimation, and Somers’ D for medians.

Covariates for adjusted analyses were selected using a 2-step procedure. First, in bivariate analysis we identified baseline covariates with an important association with the outcome (for dichotomous outcomes: *P* < .2, or RR >2.0 or <0.5; for continuous outcomes: *P* < .2, or difference >0.25 standard deviation). Next, selected covariates were entered in a multivariable regression model using a forward stepwise selection procedure. In both models, we included season of birth, because it influences exposure to wild-type rotavirus, and IYCF, to account for the other randomization in the factorial trial design.

A per-protocol analysis examined the impact of WASH on RVV immunogenicity when behavior-change modules were delivered at high fidelity [15]. We also undertook several sensitivity analyses. First, we excluded children who were seropositive prevaccination and presumed to have acquired wild-type rotavirus. Second, we restricted timing of immunogenicity measurements to tighter windows, which we defined as 0–14 days before the first dose of vaccine (for prevaccine titer) and 21–60 days after the last dose of vaccine (for postdose titer), based on previous studies [24]. Finally, a prespecified subgroup analysis by infant sex was planned if the interaction terms for the primary outcome were statistically significant.

All statistical analyses were performed using Stata version 14 (StataCorp, College Station, Texas) and Prism version 7 (GraphPad Software, La Jolla, California) software.

## RESULTS

### Baseline Characteristics

**Figure 1.**
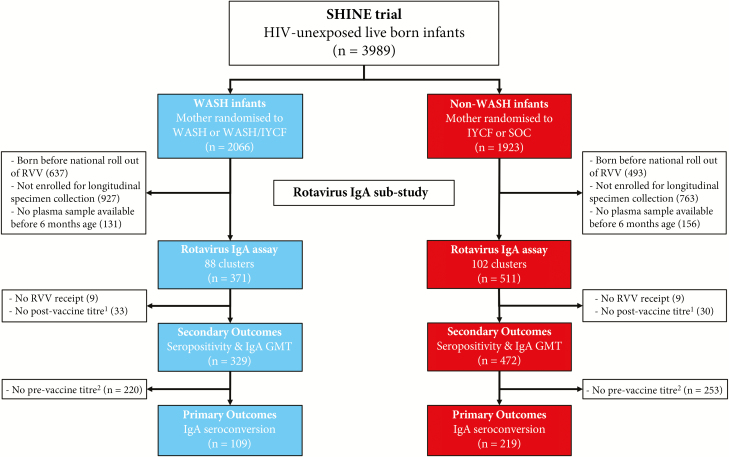
Consolidated Standards of Reporting Trials (CONSORT) flow diagram for the Sanitation Hygiene Infant Nutrition Efficacy (SHINE) rotavirus substudy. The CONSORT diagram for the full trial, with additional detail, can be found in Supplementary Figure 1. Abbreviations: GMT, geometric mean titer; HIV, human immunodeficiency virus; IgA, immunoglobulin A; IYCF, infant and young child feeding; RVV, rotavirus vaccine; SOC, standard of care; WASH, water, sanitation, and hygiene. ^a^No sample available, insufficient postvaccine sample or assay failure. ^b^Insufficient or no prevaccine sample available.

Among 5280 enrolled mothers, there were 3989 HIV-unexposed live births (Supplementary Figure 1). Of these, 882 fulfilled the inclusion criteria for this substudy and had antirotavirus IgA measured; 81 were subsequently excluded from analysis because they had no record of RVV receipt (n = 18) or no postvaccine titer measurement (n = 63) (Figure 1). This analysis therefore included 801 infants (329 WASH infants from 84 clusters; 472 non-WASH infants from 102 clusters). All infants had postvaccine titers measured and were included in the secondary outcomes (seropositivity and GMT); a subset of 328 infants had both prevaccine and postvaccine titers measured and were included in the primary outcome (seroconversion) (Supplementary Figure 2). Baseline characteristics of infants and their mothers were generally well balanced (Table 1). The substudy population was largely comparable with the overall trial population of HIV-negative births, although infants in this substudy were less likely to be preterm (Supplementary Table 1).

**Table 1.** Baseline Characteristics of Households, Infants, and Their Mothers

Characteristic^a^	WASH	Non-WASH
Infant characteristics	(n = 329)	(n = 472)
Sex, female	52.9	47.0
Preterm (<37 wk)	16.7	15.8
Birthweight, kg, mean (SD)	3.1 (0.5)	3.1 (0.5)
Low birthweight (<2.5 kg)	8.5	7.2
Institutional delivery	91.0	89.0
Normal vaginal delivery	93.7	94.3
Born in rotavirus season^b^	31.6	38.8
Exclusive breastfeeding^c^	91.5	91.2
Maternal characteristics	(n = 323)	(n = 469)
Age, y, mean (SD)	27.4 (6.7)	26.5 (7.3)
Parity, median (IQR)	2 (1–3)	2 (1–3)
Height, cm, mean (SD)	159.8 (5.3)	160.3 (5.8)
MUAC, cm, mean (SD)	26.9 (3.6)	26.6 (2.9)
Married	94.3	95.6
Completed years of schooling, median (IQR)	10 (9–11)	10 (9–11)
Unemployed	86.4	92.0
Religion		
Apostolic	44.7	46.8
Other Christian	47.8	48.2
Other religion	7.6	5.0
Wealth quintile		
Lowest	15.8	15.8
Second	21.7	19.6
Middle	21.7	19.8
Fourth	19.8	21.3
Highest	18.9	19.2
Electricity	1.0	4.3
Other electric power		
Generator	4.1	1.6
Solar power	74.8	70.9
None	21.1	27.5
Household characteristics		
Household size, median (IQR)	5 (4–6)	5 (3–6)
Sanitation		
Open defecation	43.1	52.7
Any latrine^d^	49.2	36.7
Improved latrine^d^	46.6	30.0
Improved latrine with trodden path	29.9	26.7
Water		
Improved water	65.5	62.9
Treat water	12.1	10.9
Time to drinking water, min, median (IQR)	10 (5–15)	7 (5–15)
Per capita water volume, L, mean (SD)	9.0 (7.6)	9.2 (7.6)
Hygiene		
Handwashing station present^d^	22.3	2.6
Handwashing station filled with water	7.5	0.5
Improved floor	57.1	53.7
No. of chickens, median (IQR)	6 (2–12)	6 (2–11)
Livestock observed in house	44.4	40.4
Feces observed in yard	36.3	33.8

Data are presented as percentage unless otherwise indicated.

Abbreviations: IQR, interquartile range; MUAC, mean upper arm circumference; SD, standard deviation; WASH, water, sanitation, and hygiene.

^a^Baseline for maternal and household characteristics was 2 weeks after consent (~14 weeks’ gestation); baseline for infants was at birth.

^b^Rotavirus season in Zimbabwe defined as 1 April–31 July.

^c^Assessed at 3 months of age.

^d^Median enrollment was at 12.1 weeks’ gestation and the median baseline visit at 16.4 weeks’ gestation. In some instances, WASH hardware was therefore introduced before the baseline visit, leading to an apparent imbalance in household WASH characteristics between arms.

### Intervention Delivery and Uptake

There was high fidelity of intervention delivery (Supplementary Table 2). Among WASH households, 98.4% received latrines, 100% received hand-washing stations, and 96.8% received ≥80% of planned soap deliveries. VHWs completed >95% of planned behavior-change visits. Participant uptake of interventions, measured as observed and reported behaviors at the 3-month postnatal visit, was high (Supplementary Table 2).

### Rotavirus Vaccination

All 801 children had documented receipt of at least 1 dose of RVV, and 789 (98.5%) had documented receipt of 2 doses. The median age at rotavirus vaccination was 6.3 (interquartile range [IQR], 6.0–7.0) weeks for dose 1 and 11.0 (IQR, 10.3–12.5) weeks for dose 2, with no evidence of difference between WASH and non-WASH groups (Table 2). The median timing of prevaccine titer measurement was 10 (IQR, 7–16) days prior to the first dose of RVV, and for postvaccine titer measurement was 28 (IQR 19–47) days after the last dose of RVV, with no evidence of difference between groups (Table 2). Among 328 infants included in the primary outcome, 2 of 109 (1.8%) in the WASH group vs 15 of 219 (6.8%) in the non-WASH group were seropositive at baseline (*P* = .126; Table 2).

**Table 2.** Rotavirus Vaccination in Water, Sanitation, and Hygiene (WASH) and Non-WASH Groups

Vaccination	WASH Infants	Non-WASH Infants	*P* Value^a^
Age at Rotarix dose 1, days, median (IQR)	44 (42–49)	44 (42–48)	.889
Age at Rotarix dose 2, days, median (IQR)	77 (72–88)	77 (72–87)	.292
Timing of predose titer measurement, days prior to first vaccine dose, median (IQR)^b^	–10 (–16 to –7)	–10 (–15 to –7)	.760
Timing of postdose titer, days after second vaccine dose, median (IQR)^c^	29 (20–51)	28 (19–44)	.141
Baseline rotavirus IgA seropositive^d^, %	1.8	6.8	.126

Abbreviations: IgA, immunoglobin A; IQR, interquartile range.

a*P* values adjusted for clustering effect. Depending on the analysis, other methods for comparing arms while handling within-cluster correlation included multinomial and ordinal regression models with robust variance estimation, and Somers’ D for medians, all implemented in Stata version 14.

bPredose titer only available in 109 WASH infants and 219 non-WASH infants.

cAfter the last dose of rotavirus vaccine.

dBaseline seropositivity/seronegativity refers to rotavirus immunoglobulin A titer measurements prevaccine. Numbers are therefore based only on infants with available prevaccine titers (n = 328).

### Primary Outcome

**Figure 2.**
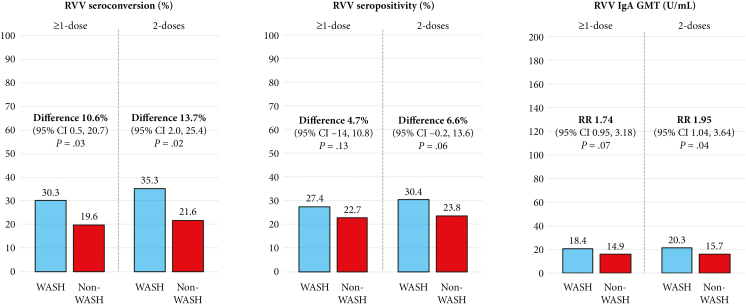
Primary outcome (rotavirus vaccine seroconversion) and secondary outcomes (rotavirus vaccine seropositivity and immunoglobulin A geometric means titers) in water, sanitation, and hygiene (WASH) and non-WASH groups. Results shown for both infants who received at least 1 dose of rotavirus vaccine and infants who received 2 doses of rotavirus vaccine. Abbreviations: CI, confidence interval; GMT, geometric mean titer; IgA, immunoglobulin A; RR, relative risk; RVV, rotavirus vaccine.

In the WASH group, 33 of 109 (30.3%) infants seroconverted following rotavirus vaccination, compared to 43 of 219 (19.6%) in the non-WASH group (absolute difference, 10.6% [95% confidence interval {CI}, .5%–20.7%]; *P* = .031; Figure 2). Among infants who received both doses of RVV, 30 of 85 (35.3%) in the WASH group vs 41 of 190 (21.6%) in the non-WASH group seroconverted (absolute difference, 13.7% [95% CI, 2.0%–25.4%]; *P* = .016; Figure 2). Findings remained similar after adjustment and in the per-protocol analysis (Supplementary Table 3).

### Secondary Outcomes

In the WASH group, 90 of 329 (27.4%) infants were seropositive following RVV, compared to 107 of 472 (22.7%) in the non-WASH group (absolute difference, 4.7% [95% CI, –1.4% to 10.8%]; *P* = .130; Figure 2). There was weak evidence of higher postvaccination antirotavirus IgA GMTs among infants in the WASH group compared to the non-WASH group (18.4 [95% CI, 15.6–21.7] U/mL vs 14.9 [95% CI, 13.2–16.8] U/mL, respectively; absolute difference, 1.74 [95% CI, .95–3.18] U/mL; *P* = .072; Figure 2). Among infants who received both doses of RVV, differences between groups were marginally increased (Figure 2); findings remained similar after adjustment and in the per-protocol analysis (Supplementary Table 4).

### Sensitivity Analyses

We conducted sensitivity analyses to account for variation in the timing of pre- and postvaccine titer measurements, and for baseline seropositivity due to wild-type rotavirus infection. Among 142 infants who had prevaccine and postvaccine titers measured within a more restricted window, 15 of 48 (31.3%) in the WASH group compared to 19 of 94 (20.2%) in the non-WASH group seroconverted (absolute difference, 11.0% [95% CI, –4.4% to 26.5%]; *P* = .107; Supplementary Table 5). After excluding 17 of 328 (5.2%) infants who were seropositive prior to vaccination, inferences remained unchanged: 33 of 107 (30.8%) in the WASH group vs 43 of 204 (21.1%) in the non-WASH group seroconverted following rotavirus vaccination (absolute difference, 9.8% [95% CI, –.6% to 20.2%]; *P* = .057; Supplementary Table 6). Finally, in a prespecified subgroup analysis, there was no significant interaction between infant sex and RVV seroconversion (*P* = .52).

## DISCUSSION

Improvements in household WASH in rural Zimbabwe increased infant seroconversion to RVV. Among infants in the non-WASH group, approximately 20% seroconverted to the RVV, compared to >30% in the WASH group. Both the direction and magnitude of effect remained highly consistent after adjustment and in multiple sensitivity analyses. Together, these findings suggest that environmental health improvements could help to increase oral vaccine immunogenicity. However, our findings also highlight the poor seroconversion to RVV even after substantial household WASH improvements, showing that other approaches will be required to realize the full benefits of oral vaccination in developing countries.

Our finding that WASH modestly increased RVV seroconversion provides some insight into the mechanisms underlying poor responses to RVV. It is plausible that conditions of poor WASH perturb the intestinal milieu, thereby reducing the immunogenicity of oral vaccines [6]. For example, carriage of enteropathogens has been associated with reduced immune responses to RVV [25]. Environmental enteric dysfunction may impair oral vaccine processing during transit through the small intestine, although its contribution to oral vaccine underperformance remains uncertain [26]. Some studies have found differences in the composition of the intestinal microbiota between responders and nonresponders to RVV [9]. The WASH intervention may therefore confer benefits for oral vaccine immunogenicity via several mechanisms. Future data on enteropathogen carriage and the microbiota from SHINE will provide important mechanistic insights. Alternatively, WASH may confer benefits for oral vaccination by reducing exposure to wild-type rotavirus infection. Fewer seropositive infants at baseline in the WASH group would increase susceptibility to vaccination as the first exposure to rotavirus. Nonetheless, after excluding baseline seropositive infants, our inferences remained unchanged, suggesting that this is not the only mechanism underlying our findings.

Numerous trials have explored interventions to augment oral vaccine immunogenicity. Some have aimed to promote gut health and mucosal responsiveness, including probiotics [27], antibiotics [28], and zinc [29], but overall these have shown no effect [30]. Changes to the formulation or schedule of oral vaccines are the only strategies with evidence of benefit for oral vaccine immunogenicity [30]. The modest effect size from the WASH intervention in our study is comparable with reported effect sizes following alterations in vaccine scheduling. However, WASH has the advantage of already being a priority intervention to reach Sustainable Development Goal targets, whereas changes to vaccination schedules may be difficult to implement. Whether the finding of increased seroconversion with WASH translates into improved vaccine efficacy cannot be addressed in the current study. However, a recent observational study in Malawi showed that reductions in all-cause mortality following RVV introduction were greater in areas also receiving intensive sanitation improvements, suggesting potential synergy between interventions [31].

The promising difference in vaccine responses with WASH is an important proof of concept but is tempered by the scale of oral vaccine underperformance. RVV seroconversion in the WASH group was 39% at best (Supplementary Table 5), which is still far lower than in developed countries (67%–98%) [5]. It is, however, consistent with recent data from other developing countries: RVV seroconversion was 24% in Malawi [32] and 27% in Bangladesh [33]. Taken together, there are important implications of these findings. First, our WASH interventions may have been insufficiently effective and further improvements in vaccine immunogenicity may be gained through more transformative WASH approaches. The household-level elementary WASH interventions implemented in SHINE were similar to those commonly accessible in rural areas of developing countries. However, we have recently shown that these interventions had no impact on stunting, anemia, or diarrhea [15], similar to other trials [34, 35]. We have therefore argued that more effective approaches may be required to achieve the desired public health benefits of improved WASH. Alternatively, factors not addressed by WASH may contribute to the immunogenicity gap, such as genetic variation [36], interference from maternal antibodies, and micronutrient deficiencies. Other approaches that address these factors may be required.

This is the first study to explore the impact of a WASH intervention on oral vaccine responses, leveraging the cluster-randomized design of SHINE and the introduction of RVV in Zimbabwe. There was high reported uptake of WASH interventions, which were based on extensive formative research. We prespecified all outcomes and ran laboratory assays and statistical analyses blind to intervention arm to reduce bias. However, there are some important limitations. First, SHINE was not a vaccine trial and we therefore did not control vaccine administration or timing of pre- and postvaccine titer measurements. This may have contributed to the overall lower rates of seroconversion than is typical of published RVV trials in southern Africa [22]. However, >98% of infants received at least 1 dose of RVV and the median time-point of immunogenicity measurement was 28 days postvaccination, with no significant difference between WASH and non-WASH groups. In addition, when we restricted analyses to infants who had titers measured during a narrower window, our inferences remained unchanged. Second, although we exceeded our sample size, the final number of infants in the primary outcome analysis (which required both prevaccine and postvaccine plasma samples) was smaller than planned and the precision of our seroconversion estimates was likely reduced. Finally, antirotavirus IgA is a limited correlate of protection from disease [24], particularly in developing countries [33]. Nevertheless, it remains the preferred measure of RVV response and is likely to be biologically meaningful given the enormous differences in seroconversion rates between developing and developed countries [5].

Oral rotavirus vaccination has substantially reduced diarrheal mortality globally [37]; however, its underperformance in developing countries limits its full potential. Improvements in the efficacy of RVV would confer major public health benefits. We show here that improvements in household WASH significantly increased RVV seroconversion. However, the increase was modest and seroconversion remained low overall, showing the enormous challenge of oral vaccine underperformance. More effective WASH interventions and alternative strategies are likely required to further improve the performance of oral vaccines so that all children in developing countries can benefit from their impact.

## Supplementary Data

Supplementary materials are available at *Clinical Infectious Diseases* online. Consisting of data provided by the authors to benefit the reader, the posted materials are not copyedited and are the sole responsibility of the authors, so questions or comments should be addressed to the corresponding author.

## Supplementary Material

ciz140_suppl_Supplementary_Material

## Notes

***Disclaimer.***The study funders approved the trial design, but were not involved in data collection, analysis, interpretation, or manuscript preparation. The corresponding author had full access to the data and made the decision to submit for publication.

***Author contributions.***J. A. C., J. H. H., and A. J. P. designed the substudy. J. A. C., B. L., M. P. C., M. M., and B. D. K. developed the laboratory techniques. J. A. C., M. G., S. R., and K. M. performed the laboratory assays. N. V. T. managed field operations and F. D. M. supervised all data collection in the trial. J. A. C., B. C., R. N., L. M., and A. J. P. analyzed and interpreted the data. J. A. C. wrote the first draft of the manuscript. All authors were involved in subsequent revisions of the manuscript for important intellectual content and have read and approved this final version to be published.

***Acknowledgments.***The authors thank the mothers and babies who participated in the SHINE trial, and the SHINE Trial Team, members of whom are listed at https://doi.org/10.1093/cid/civ844. They acknowledge the leadership and staff of the Ministry of Health and Child Care in Chirumanzu and Shurugwi districts and Midlands province (especially environmental health, nursing, and nutrition) for their roles in operationalization of the study procedures. They also acknowledge the Ministry of Local Government officials in each district who supported and facilitated field operations. They thank Philippa Rambanepasi and her team for proficiently managing finances and Virginia Sauramba for managing compliance issues. They also thank Dr Sean Diehl at the University of Vermont for his help in establishing the rotavirus assay.

***Financial support.***This work was supported by the Wellcome Trust (grant numbers 203905/Z/16/Z to J. A. C. and 093768/Z/10/Z and 108065/Z/15/Z to A. J. P.). The SHINE trial was funded by the Bill & Melinda Gates Foundation (grant numbers OPP1021542 and OPP113707); the UK Department for International Development (UK Aid); the Swiss Agency for Development and Cooperation (SDC); and the National Institutes of Health (NIH) (grant numbers 2R01HD060338-06).

***Potential conflicts of interest.***All authors: No reported conflicts. All authors have submitted the ICMJE Form for Disclosure of Potential Conflicts of Interest. Conflicts that the editors consider relevant to the content of the manuscript have been disclosed.

## References

[1] Tate JE, Burton AH, Boschi-Pinto C, Parashar UD; World Health Organization–Coordinated Global Rotavirus Surveillance Network Global, regional, and national estimates of rotavirus mortality in children <5 years of age, 2000-2013. Clin Infect Dis 2016; 62(Suppl 2):S96–105. 27059362 10.1093/cid/civ1013 PMC11979873

[2] International Vaccine Access Center. IVAC. 2017. Available at: http://www.view-hub.org/viz/.

[3] Vesikari T, Matson DO, Dennehy P, et al; Rotavirus Efficacy and Safety Trial (REST) Study Team Safety and efficacy of a pentavalent human-bovine (WC3) reassortant rotavirus vaccine. N Engl J Med 2006; 354:23–33. 16394299 10.1056/NEJMoa052664

[4] Armah GE, Sow SO, Breiman RF, et al. Efficacy of pentavalent rotavirus vaccine against severe rotavirus gastroenteritis in infants in developing countries in sub-Saharan Africa: a randomised, double-blind, placebo-controlled trial. Lancet 2010; 376:606–14. 20692030 10.1016/S0140-6736(10)60889-6

[5] Patel M, Glass RI, Jiang B, Santosham M, Lopman B, Parashar U A systematic review of anti-rotavirus serum IgA antibody titer as a potential correlate of rotavirus vaccine efficacy. J Infect Dis 2013; 208:284–94. 23596320 10.1093/infdis/jit166

[6] Parker EP, Ramani S, Lopman BA, et al. Causes of impaired oral vaccine efficacy in developing countries. Future Microbiol 2018; 13:97–118. 29218997 10.2217/fmb-2017-0128 PMC7026772

[7] Lagos R, Fasano A, Wasserman SS, et al. Effect of small bowel bacterial overgrowth on the immunogenicity of single-dose live oral cholera vaccine CVD 103-HgR. J Infect Dis 1999; 180:1709–12. 10515838 10.1086/315051

[8] Posey DL, Linkins RW, Oliveria MJ, Monteiro D, Patriarca PA The effect of diarrhea on oral poliovirus vaccine failure in Brazil. J Infect Dis 1997; 175(Suppl 1):S258–63. 9203726 10.1093/infdis/175.supplement_1.s258

[9] Harris VC, Armah G, Fuentes S, et al. Significant correlation between the infant gut microbiome and rotavirus vaccine response in rural Ghana. J Infect Dis 2017; 215:34–41. 27803175 10.1093/infdis/jiw518 PMC5225256

[10] Campbell DI, Murch SH, Elia M, et al. Chronic T cell-mediated enteropathy in rural West African children: relationship with nutritional status and small bowel function. Pediatr Res 2003; 54:306–11. 12788978 10.1203/01.PDR.0000076666.16021.5E

[11] Becker-Dreps S, Vilchez S, Bucardo F, et al. The association between fecal biomarkers of environmental enteropathy and rotavirus vaccine response in Nicaraguan infants. Pediatr Infect Dis J 2017; 36:412–6. 27977553 10.1097/INF.0000000000001457

[12] Naylor C, Lu M, Haque R, et al; PROVIDE Study Teams Environmental enteropathy, oral vaccine failure and growth faltering in infants in Bangladesh. EBioMedicine 2015; 2:1759–66. 26870801 10.1016/j.ebiom.2015.09.036 PMC4740306

[13] Church J.A., Parker E, Kirkpatrick B, Grassly N, Prendergast AJ. Interventions to improve oral vaccine performance: a systematic review and meta-analysis. Lancet Infect Dis, 2019;19:203–14. 10.1016/S1473-3099(18)30602-9 PMC6353819 30712836

[14] Sanitation Hygiene Infant Nutrition Efficacy Trial Team; Humphrey JH, Jones AD, Manges A, et al The Sanitation Hygiene Infant Nutrition Efficacy (SHINE) trial: rationale, design, and methods. Clin Infect Dis 2015; 61(Suppl 7): S685–702. 26602296 10.1093/cid/civ844 PMC4657589

[15] Humphrey JH, Mbuya MNN, Ntozini R, et al; Sanitation Hygiene Infant Nutrition Efficacy (SHINE) Trial Team Independent and combined effects of improved water, sanitation, and hygiene, and improved complementary feeding, on child stunting and anaemia in rural Zimbabwe: a cluster-randomised trial. Lancet Glob Health 2019; 7:e132–47. 30554749 10.1016/S2214-109X(18)30374-7 PMC6293965

[16] Morgan PR A ventilated pit privy. Appropriate Technol 1979; 6:10–11.

[17] Watt J The Tippy Tap: a simple handwashing device for rural areas. J Trop Pediatr 1988; 34:91–2. 3385859 10.1093/tropej/34.2.91

[18] Prendergast AJ, Humphrey JH, Mutasa K, et al; Sanitation Hygiene Infant Nutrition Efficacy (SHINE) Trial Team Assessment of environmental enteric dysfunction in the SHINE trial: methods and challenges. Clin Infect Dis 2015; 61(Suppl 7):S726–32. 26602300 10.1093/cid/civ848 PMC4657593

[19] United Nations Children’s Fund. Zimbabwe: WHO and UNICEF estimates of immunization coverage: 2016 revision. https://data.unicef.org/wp-content/uploads/country_profiles/Zimbabwe/immunization_country_profiles/immunization_zwe.pdf. Access 1 July 2018.

[20] Ward RL, Bernstein DI, Shukla R, et al. Effects of antibody to rotavirus on protection of adults challenged with a human rotavirus. J Infect Dis 1989; 159:79–88. 2535868 10.1093/infdis/159.1.79

[21] Bernstein DI, Smith VE, Sherwood JR, et al. Safety and immunogenicity of live, attenuated human rotavirus vaccine 89-12. Vaccine 1998; 16:381–7. 9607059 10.1016/s0264-410x(97)00210-7

[22] Madhi SA, Cunliffe NA, Steele D, et al. Effect of human rotavirus vaccine on severe diarrhea in African infants. N Engl J Med 2010; 362:289–98. 20107214 10.1056/NEJMoa0904797

[23] Moulton LH Covariate-based constrained randomization of group-randomized trials. Clin Trials 2004; 1:297–305. 16279255 10.1191/1740774504cn024oa

[24] Angel J, Steele AD, Franco MA Correlates of protection for rotavirus vaccines: possible alternative trial endpoints, opportunities, and challenges. Hum Vaccin Immunother 2014; 10:3659–71. 25483685 10.4161/hv.34361 PMC4514048

[25] Taniuchi M, Platts-Mills JA, Begum S, et al. Impact of enterovirus and other enteric pathogens on oral polio and rotavirus vaccine performance in Bangladeshi infants. Vaccine 2016; 34:3068–75. 27154394 10.1016/j.vaccine.2016.04.080 PMC4912219

[26] Church JA, Parker EP, Kosek MN, et al. Exploring the relationship between environmental enteric dysfunction and oral vaccine responses. Future Microbiol 2018; 13:1055–70. 29926747 10.2217/fmb-2018-0016 PMC6136084

[27] Zimmermann P, Curtis N The influence of probiotics on vaccine responses—a systematic review. Vaccine 2018; 36:207–13. 28923425 10.1016/j.vaccine.2017.08.069

[28] Grassly NC, Praharaj I, Babji S, et al. The effect of azithromycin on the immunogenicity of oral poliovirus vaccine: a double-blind randomised placebo-controlled trial in seronegative Indian infants. Lancet Infect Dis 2016; 16:905–14. 27156189 10.1016/S1473-3099(16)30023-8

[29] Lazarus RP, John J, Shanmugasundaram E, et al. The effect of probiotics and zinc supplementation on the immune response to oral rotavirus vaccine: a randomized, factorial design, placebo-controlled study among Indian infants. Vaccine 2018; 36:273–9. 28874323 10.1016/j.vaccine.2017.07.116 PMC12001858

[30] Church JA, Parker E, Kirkpatrick B, Grassly B, Prendergast AJ Interventions to improve oral vaccine performance: a systematic review and meta-analysis. Lancet Infect Dis 2019; 19:203–14. 30712836 10.1016/S1473-3099(18)30602-9 PMC6353819

[31] Bar-Zeev N, King C, Phiri T, et al; VacSurv Consortium Impact of monovalent rotavirus vaccine on diarrhoea-associated post-neonatal infant mortality in rural communities in Malawi: a population-based birth cohort study. Lancet Glob Health 2018; 6:e1036–44. 30103981 10.1016/S2214-109X(18)30314-0 PMC6088152

[32] Pollock L, Bennett A, Jere KC, et al Non-secretor histo-blood group antigen phenotype is associated with reduced risk of clinical rotavirus vaccine failure in Malawian infants [manuscript published online ahead of print 18 December 2018]. Clin Infect Dis 2018. doi:10.1093/cid/ciy1067. PMC6763638 30561537

[33] Lee B, Carmolli M, Dickson DM, et al Rotavirus-specific IgA responses are impaired and serve as a suboptimal correlate of protection among infants in Bangladesh. Clin Infect Dis 2018; 67:186–92. 29394355 10.1093/cid/ciy076 PMC6030840

[34] Null C, Stewart CP, Pickering AJ, et al. Effects of water quality, sanitation, handwashing, and nutritional interventions on diarrhoea and child growth in rural Kenya: a cluster-randomised controlled trial. Lancet Glob Health 2018; 6:e316–29. 29396219 10.1016/S2214-109X(18)30005-6 PMC5809717

[35] Luby SP, Rahman M, Arnold BF, et al. Effects of water quality, sanitation, handwashing, and nutritional interventions on diarrhoea and child growth in rural Bangladesh: a cluster randomised controlled trial. Lancet Glob Health 2018; 6:e302–15. 29396217 10.1016/S2214-109X(17)30490-4 PMC5809718

[36] Kazi AM, Cortese MM, Yu Y, et al. Secretor and salivary ABO blood group antigen status predict rotavirus vaccine take in infants. J Infect Dis 2017; 215:786–9. 28329092 10.1093/infdis/jix028

[37] Platts-Mills JA, Steele AD Rotavirus vaccine impact in Africa: greater than the sum of its parts? Lancet Glob Health 2018; 6:e948–9. 30103987 10.1016/S2214-109X(18)30356-5

