# Supplementary material for: The Impact of Improved Water, Sanitation, and Hygiene on Oral Rotavirus Vaccine Immunogenicity in Zimbabwean Infants: Substudy of a Cluster-randomized Trial
Source: Clin Infect Dis. 2019 Mar 29;69(12):2074–81. doi: 10.1093/cid/ciz140 (PMC6880336; doi:10.1093/cid/ciz140)

The impact of improved water, sanitation and hygiene on oral rotavirus vaccine immunogenicity in Zimbabwean infants: sub-study of a cluster-randomized trial

James A Church, Sandra Rukobo, Margaret Govha, Benjamin Lee, Marya P Carmolli, Bernard Chasekwa, Robert Ntozini, Kuda Mutasa, Monica M McNeal, Florence D. Majo, Naume V. Tavengwa, Lawrence H Moulton, Jean H Humphrey, Beth D Kirkpatrick, Andrew J Prendergast

Table of Contents for Appendix

**Supplementary methods**		
i.	SHINE trial randomization procedure	Page 2
ii.	Further details of SHINE interventions	Page 3
iii.	Definition of per protocol population	Page 4
**Supplementary tables**		
i.	Table S1: Baseline characteristics between sub-study enrolled and not-enrolled	Page 5
ii.	Table S2: WASH intervention delivery & uptake	Page 6
iii.	Table S3: Primary outcome (ITT & PP analysis)	Page 7
iv.	Table S4: Secondary outcomes (ITT & PP analysis)	Page 8
v.	Table S5: Sensitivity analysis #1 (restricted window of pre- and post-vaccine titre)	Page 10
vi.	Table S6: Sensitivity analysis #2 (excluding children seropositive at baseline)	Page 11
**Supplementary figures**		
i.	Figure S1: Detailed CONSORT flow diagram	Page 12
ii.	Figure S2: Scatter plot of IgA titres (WASH vs non-WASH)	Page 13

Supplementary methods

i. SHINE trial randomization procedure

A highly constrained randomization technique was used to allocate clusters (stratified by district) to treatments. We randomly selected 1000 allocations from among 5000 computer-generated allocations that balanced the 4 treatment arms on 14 parameters without pre-specified bounds. From the 1000, we randomly selected 10 for a public randomization ceremony; Figure A. Each randomization scheme divided the randomization units into 4 groups of approximately 53 units. Each scheme's corresponding colour-coded map was printed on a separate sheet and displayed at a public forum attended by all elected councillors from the study area, District and Provincial Administrators, and Ministry of Health and Child Care authorities. In their presence, 10 plastic balls (numbered 1-10) were placed in an opaque sack. A community representative selected one ball from the sack, thereby identifying which of the 10 numbered allocations would be used. Then, four balls (labelled A, B, C, and D) were placed in one sack, and four balls (labelled with the 4 treatment arms) were placed in a second sack. Representatives drew a ball from the first sack and a ball from the second sack, pairing a group of clusters with one of the four treatment arms, thereby mimicking a widely known World Cup draw procedure. This was repeated twice more to pair the next two groups of clusters with two more treatment arms. The remaining balls formed the final pairing. This second stage was included to provide an additional assurance of impartiality/randomness and a further opportunity for participation of the community leadership.

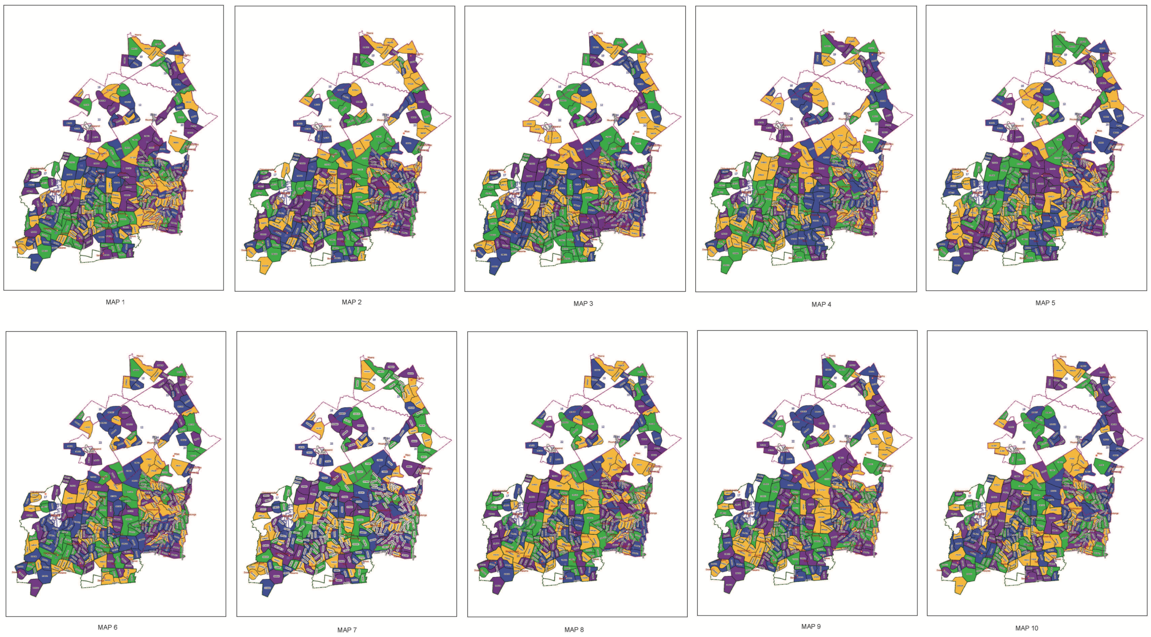

Figure A. Maps of ten SHINE randomization schemes

ii. Further details of SHINE interventions

Standard of Care (SOC) Intervention: Village Health Workers were trained through the Ministry of Health and Child Care curriculum, which instructs VHWs to visit pregnant women and infants frequently, although the precise content or purpose of each visit is not specified. Consequently the SHINE SOC intervention was designed to standardize the number of visits (3 antenatal and 12 postnatal visits) and the content of primary health care messages across treatment arms. Four of these visits promoted exclusive breastfeeding (EBF) from birth to 6 months using modules designed to overcome contextual barriers identified in formative work. Other SOC modules include prevention of mother-to-child HIV transmission (PMTCT), antenatal care, hospital-based delivery, family planning and immunizations.

WASH Intervention: Within 6 weeks of enrolment (~20 weeks gestation) into the WASH and WASH+IYCF arms of the trial, a Blair Ventilated Improved Pit (VIP) Latrine was constructed at the participant's household and two 'Tippy Tap' hand-washing stations (locally manufactured, and adapting the model piloted by the Kenya WASH Benefits trial) were installed near the latrine and kitchen; Figure B.

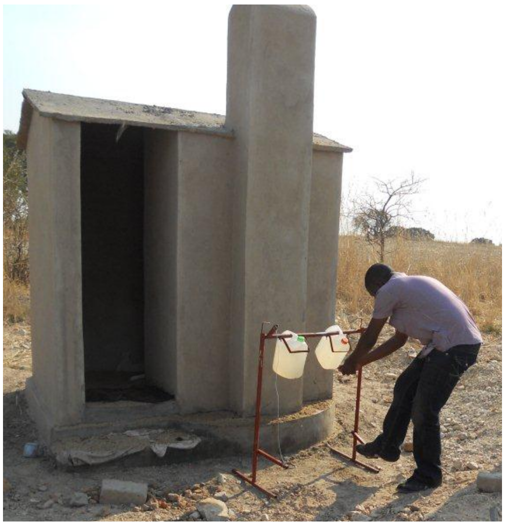

Figure B: Blair VIP latrine and Tippy Tap

WASH Modules 1 (delivered at 24 gestational weeks) and 2 (32 gestational weeks) promoted safe disposal of faeces, and hand-washing with soap after faecal contact and before food preparation and eating, respectively. Our intention was for the baby to be born into a household in which latrine use and household hand-washing behaviours were normalised and habitual. WASH Module 3 (protecting babies from faecal ingestion during exploratory play) was delivered when the baby was 2 months old; a washable 2.8m x 3.0m locally manufactured mat and plastic play yard (North States, Minneapolis MN) were provided at 2 months and 6 months, respectively; Figure C.

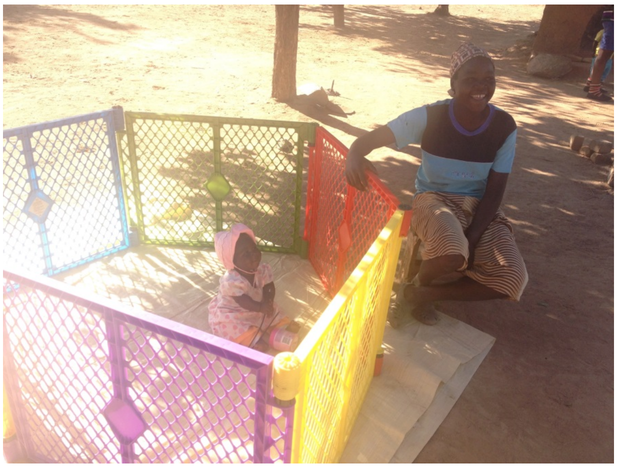

Figure C: SHINE mat and play yard

WASH Module 4 (treat all drinking water given to babies after 6 months of EBF) was delivered at 4 months of age, along with point-of-use chlorination (WaterGuard: a dilute sodium hypochlorite solution, manufactured locally by Nelspot). Liquid soap and Water Guard were regularly replenished from time of introduction (Module 2 and 5, respectively) until the infant was 18 months old. WASH Module 5, delivered at 5 months of age, stressed the importance of freshly preparing or fully reheating all foods fed to infants. A review module was delivered at 12 months.

IYCF Intervention: IYCF Module 1 (delivered at 5 months) linked good infant feeding to child growth, health, and intelligence. IYCF Module 2 (6 months) promoted feeding nutrient-dense food, including 20 g per day of the lipid-based nutrient supplement (LiNS) developed by the International Lipid-Based Nutrients Supplements Project, provided monthly when the baby was 6 to 18 months of age. Module 3 (7 months) was a participatory cooking demonstration in which any available household food was prepared and fed to the baby, stressing three messages from formative research: 1) an infant can eat any food that an adult eats; 2) food should be ground so that the infant can swallow and digest it; 3) food that is locally available is important for the infant. Module 4 (8 months) promoted responsive feeding during illness, Module 5 (9 months) promoted diet diversity, and a review module was delivered at 12 months.

iii. Definition of per-protocol population

The per-protocol analysis examined the impact of the interventions when behaviour-change modules were delivered at high fidelity (pre-defined for the WASH+IYCF group as receiving all 6 core modules schedule up until 6 months postnatal; for other study arms, pre-defined as receiving all modules scheduled at the same time-points when WASH+IYCF core modules were delivered).

Supplementary tables

Table S1: Comparison of baseline characteristics between HIV negative mothers and their infants enrolled into the rotavirus sub-study and those not enrolled. (Live births only; 3937 mothers and 3989 infants).

	**Enrolled into substudy** (Mothers N=790) (Infants/babies N=801)	**Not enrolled into substudy** (Mothers N=3147) (Infants/babies N=3188)	**P value**
Household size median (IQR)	5 (4,6)	5 (3,6)	0.569
Any latrine, %	40.5	40.2	0.845
Improved water, %	63.4	63.2	0.913
Hand washing station present, %	11.3	8.2	<0.001
**Infant characteristics**			
Gender, % female	49.4	49.4	0.711
Preterm (<37weeks), %	16.0	24.8	<0.001
Birthweight, kg; mean (SD)	3.13 (0.49)	3.09 (0.53)	<0.001
Low birthweight (<2.5kg), %	7.7	8.2	<0.001
Institutional delivery, %	89.8	88.6	0.361
Normal vaginal delivery, %	94.1	92.5	0.224
Born during rotavirus season, %[1]	35.8	33.4	0.199
Exclusive breastfeeding, %[2]	91.3	87.1	0.004
**Maternal characteristics**			
Age, years; mean (SD)	26.9 (7.0)	25.3 (7.7)	<0.001
Parity; median (IQR)	2 (1, 3)	2 (1, 3)	<0.001
Height, cm; mean (SD)	160.1 (5.7)	160.1 (5.5)	<0.001
Mean upper arm circumference, cm; mean (SD)	26.8 (3.2)	26.3 (3.2)	<0.001
Married, %	95.1	95.5	0.618
Completed years of schooling, median (IQR)	10 (9,11)	10 (9,11)	0.997
Unemployed, %	89.7	92.0	0.083
Religion:			0.026
Apostolic, %	45.8	47.4	
Other Christian, %	48.1	44.2	
Other religion, %	6.1	8.4	
Wealth Quintile:			0.272
Lowest, %	15.8	17.5	
Second, %	20.5	17.5	
Middle, %	20.6	18.3	
Fourth, %	20.6	19.5	
Highest, %	19.0	19.3	
Electricity, %yes [n]	2.7	2.7	0.773
Other electric power:			0.009
Generator, %	2.6	3.4	
solar power, %	72.4	66.7	
no other type, %	24.9	30.0	
**Household characteristics**			
Household size; median (IQR)	5 (4,6)	5 (3,6)	0.736
Sanitation			
Open defecation, %	48.7	48.4	0.240
Any latrine, %	41.1	40.0	0.581
Improved latrine, %	36.9	35.2	0.377
Improved latrine with trodden path, %	28.1	28.3	0.888
Water			
Improved water, %	63.9	63.1	0.742
Treat water, %	11.5	12.9	0.301
Time to drinking water, min; median (IQR)	10 (5,15)	10 (5, 20)	<0.001
Per capita water volume, L; mean (SD)	9.3 (8.1)	9.8 (11.5)	<0.001
Hygiene			
Hand washing station present, %	10.9	8.4	0.059
Handwashing station filled with water, %	3.4	3.2	0.789
Improved floor, %	55.1	55.4	0.750
Livestock observed in house, %	41.9	35.9	0.002
Faeces observed in yard, %	34.8	30.3	0.026

[1] Rotavirus season in Zimbabwe defined as 1st April – 31st July.

[2] Assessed at 12 weeks of age

Table S2: WASH intervention delivery and participant uptake across treatment groups at the 3-month postnatal visit.[3]

Delivery of hardware, supplies and behavior change modules	Data source	WASH[4]	Non-WASH[2]	P-value[5]
Number of children[6]		313	453	
**Water sanitation and hygiene (WASH) inputs**				
SHINE-installed ventilated improved pit latrine (%)	Trial logs	98.4	N/A	
2 Hand washing stations (Tippy Taps) delivered (%)	Trial logs	100.0	N/A	
Liquid soap deliveries, median (IQR), (max=5)	Trial logs	5 (5 ; 5)	N/A	
Received ≥ 4 (≥80% of expected) soap deliveries (%)	Trial logs	96.8	N/A	
**Behavior change modules**				
Intervention modules received[7], median (IQR) (max = 7)	VHW report	7 (7 ; 7)	7 (7 ; 7)	0.060
**WASH behaviors**				
Household members who practice open defecation (%)	Maternal report	1.1	43.6	<0.001
Any latrine at household (%)	Observed	99.0	35.8	<0.001
Improved latrine at household (%)	Observed	99.0	29.9	<0.001
Improved latrine at household with well-trodden path, not used for storage, and not shared with other households (%)	Observed & maternal report	84.1	25.1	<0.001
Hand washing station at household (%)	Observed	97.1	3.1	<0.001
Hand washing station with water and rubbing agent at household (%)	Observed	80.6	1.7	<0.001
Ever treats drinking water to make it safer	Maternal report	28.4	11.2	<0.001

[3] Data are for 801 children in the rotavirus sub-study
[4] The WASH group combines the two WASH-containing arms (WASH and WASH + IYCF); the Non-WASH group combines the two arms not containing WASH (SOC and IYCF).
[5] P-values adjusted for clustering effect. Depending on the analysis, other methods for comparing arms while handling within-cluster correlation included multinomial and ordinal regression models with robust variance estimation, and Somers' D for medians
[6] Of 801 children in the rotavirus sub-study, 313 in the WASH group and 453 in the non-WASH group had uptake data available from the 3 month postnatal visit. Uptake variables were not available for 35 children due to missed visits or missing data.
[7] Partcipants across all arms received seven intervention modules before the 3 month visit, however content differed with no WASH messages in the SOC and IYCF arms.

Table S3: Primary outcome: rotavirus vaccine seroconversion in WASH and non-WASH groups.

Rotavirus vaccine seroconversion																	
**At least 1 dose rotavirus vaccine (N=328)**										**2 doses rotavirus vaccine (N=275)**							
**Analysis**	**Treatment group**	**n/N**	**%**	**Absolute difference (%) (95% CI)**	**p**	**Unadjusted**		**Adjusted[8]**		**n/N**	**%**	**Absolute difference (%) (95% CI)**	**p**	**Unadjusted**		**Adjusted[1]**	
						**Relative Risk (95% CI)**	**p**	**Relative Risk (95% CI)**	**p**					**Relative Risk (95% CI)**	**p**	**Relative Risk (95% CI)**	**p**
ITT	Non-WASH	43/219	19.6	10.6 (0.5, 20.7)	0.031	1.00 (ref)		1.00 (ref)		41/190	21.6	13.7 (2.0, 25.4)	0.016	1.0 (ref)		1.0 (ref)	
	WASH	33/109	30.3			1.48 (0.98, 2.24)	0.064	1.65 (1.12, 2.42)	0.010	30/85	35.3			1.56 (1.04, 2.34)	0.033	1.71 (1.20, 2.45)	0.003
**At least 1 dose rotavirus vaccine (N=285)**										**2 doses rotavirus vaccine (N=237)**							
PP[2]	Non-WASH	33/184	17.9	12.8 (2.2, 23.3)	0.014	1.00 (ref)				31/160	19.4	17.0 (4.6, 29.4)	0.007	1.0 (ref)			
	WASH	31/101	30.7			1.68 (1.09, 2.60)	0.019			28/77	36.4			1.83 (1.18, 2.84)	0.005		

[8] Final variables included in adjusted models were: IYCF, season of birth, prematurity, improved water, maternal age, parity, employment and religion. Covariates were selected as described in the Methods section.

Ref = reference value

Table S4: Secondary outcomes: rotavirus vaccine seropositivity and geometric mean titres in WASH and non-WASH groups.

**Rotavirus vaccine seroresponse**																	
**At least 1 dose rotavirus vaccine (N=801)**										**2 doses rotavirus vaccine (N=671)**							
**Analysis**	**Treatment group**	**n/N**	**%**	**Absolute difference (%) (95% CI)**	**p**	**Unadjusted**		**Adjusted[1]**		**n/N**	**%**	**Absolute difference (%) (95% CI)**	**p**	**Unadjusted**		**Adjusted[1]**	
						**Relative Risk (95% CI)**	**p**	**Relative Risk (95% CI)**	**p**					**Relative Risk (95% CI)**	**p**	**Relative Risk (95% CI)**	**p**
ITT	Non-WASH	107/472	22.7	4.7 (-1.4, 10.8)	0.130	1.00 (ref)		1.00 (ref)		97/408	23.8	6.6 (-0.2, 13.6)	0.057	1.0 (ref)		1.0 (ref)	
	WASH	90/329	27.4			1.20 (0.93, 1.55)	0.170	1.24 (0.96, 1.61)	0.104	80/263	30.4			1.26 (0.98, 1.63)	0.072	1.32 (1.02, 1.71)	0.034
**At least 1 dose rotavirus vaccine (N=699)**										**2 doses rotavirus vaccine (N=585)**							
PP[9]	Non-WASH	88/401	22.0	5.2 (-1.2, 11.7)	0.120	1.00 (ref)				81/347	23.3	7.3 (0.0, 14.7)	0.048	1.0 (ref)			
	WASH	81/298	27.2			1.25 (0.95, 1.64)	0.115			73/238	30.7			1.31 (1.01, 1.72)	0.045		
**Anti-Rotavirus IgA GMT (U/mL)**																	
**At least 1 dose rotavirus vaccine (N=801)**										**2 doses rotavirus vaccine (N=671)**							
**Analysis**	**Treatment group**	**N**	**Mean (95% CI)**			**Unadjusted**		**Adjusted[1]**		**N**	**Mean (95% CI)**			**Unadjusted**		**Adjusted[1]**	
						**Difference (95% CI)**	**p**	**Difference (95% CI)**	**p**					**Difference (95% CI)**	**p**	**Difference (95% CI)**	**p**
ITT	Non-WASH	472	14.9 (13.2, 16.8)			0.00 (ref)		0.00 (ref)		408	15.7 (13.7, 18.1)			0 (ref)		0 (ref)	
	WASH	329	18.4 (15.6, 21.7)			1.74 (0.95, 3.18)	0.072	1.86 (1.02, 3.41)	0.044	263	20.3 (16.8, 24.6)			1.95 (1.04, 3.64)	0.037	2.08 (1.11, 3.91)	0.023

[9] The per-protocol analysis examined the impact of the interventions when behaviour-change modules were delivered at high fidelity (pre-defined for the WASH group as receiving all 6 core modules schedule up until 6 months postnatal; for other study arms, pre-defined as receiving all modules scheduled at the same time-points when the WASH group core modules were delivered.

Ref = reference value

At least 1 dose rotavirus vaccine (N=699)										2 doses rotavirus vaccine (N=585)							
PP[2]	Non-WASH	401	14.8 (12.9, 16.9)			0.00 (ref)				347	15.6 (13.5, 18.2)			0 (ref)			
	WASH	298	18.0 (15.2, 21.3)			1.83 (0.97, 3.43)	0.060			238	20.3 (16.6, 24.8)			2.10 (1.09, 4.07)	0.028		

Table S5: Primary and secondary sub-study outcomes after restricting the window of pre- and post-vaccine titre measurement[10].

Analysis		At least 1 dose rotavirus vaccine (N=136)						2 doses rotavirus vaccine (N=117)					
	Treatment group	n/N	%	Absolute difference (%) (95% CI)	p	Unadjusted		n/N	%	Absolute difference (%) (95% CI)	p	Unadjusted	
						Relative Risk (95% CI)	p					Relative Risk (95% CI)	p
Rotavirus seroconversion	Non-WASH	21/91	23.1	10.3 (-6.0 26.5)	0.143	1.00 (ref)		21/81	25.9	13.0 (-5.5, 31.5)	0.117	1.0 (ref)	
	WASH	15/45	33.3			1.45 (0.78, 2.69)	0.236	14/36	38.9			1.48 (0.82, 2.65	0.192
At least 1 dose rotavirus vaccine (N=147)								2 doses rotavirus vaccine (N=129)					
Rotavirus seropositivity	Non-WASH	23/97	23.7	10.3 (-5.3, 25.9)	0.129	1.00 (ref)		24/90	26.7	9.2 (-8.3, 26.8)	0.198	1.0 (ref)	
	WASH	17/50	34.0			1.42 (0.81, 2.50)	0.224	14/39	35.9			1.23 (0.71, 2.11)	0.460
		N	Mean (95% CI)			Difference (95% CI)	p	N	Mean (95% CI)			Difference (95% CI)	p
Anti-rotavirus IgA GMT (U/mL)	Non-WASH	97	16.6 (12.2, 22.5)			0 (ref)		90	17.9 (12.7, 25.3)			0 (ref)	
	WASH	50	23.2 (14.4, 37.4)			2.00 (0.40 9.91)	0.297	39	19.9 (12.3, 32.2)			1.25 0.26, 5.90)	0.778

[10] We defined a narrower window as 0-14 days before the first dose of vaccine (for pre-vaccine titre) and 21-60 days after the last dose of vaccine (for post-dose titre). This was updated from the original pre-specified post-vaccine window of 7-60 days in the analysis plan, for consistency with other published rotavirus vaccine studies.

Ref = reference value

Table S6: Primary and secondary sub-study outcomes after excluding infants seropositive at baseline[11].

Analysis	At least 1 dose rotavirus vaccine (N=311)							2 doses rotavirus vaccine (N=259)					
	Treatment group	n/N	%	Absolute difference (%) (95% CI)	p	Unadjusted		n/N	%	Absolute difference (%) (95% CI)	p	Unadjusted	
						Relative Risk (95% CI)	p					Relative Risk (95% CI)	p
Rotavirus seroconversion	Non-WASH	43/204	21.1	9.8 (-0.6, 20.2)	0.057	1.0 (ref)		41/176	23.3	12.8 (0.7, 24.9)	0.031	1.0 (ref)	
	WASH	33/107	30.8			1.42 (0.94, 2.13)	0.095	30/83	36.1			1.50 (1.01, 2.24)	0.045
	At least 1 dose rotavirus vaccine (N=784)							2 doses rotavirus vaccine (N=655)					
Rotavirus seropositivity	Non-WASH	103/457	22.5	5.0 (-1.1, 11.1)	0.110	1.0 (ref)		93/394	23.6	7.0 (0.0, 14.0)	0.045	1.0 (ref)	
	WASH	90/327	27.5			1.22 (0.95, 1.57)	0.122	80/261	30.7			1.29 (1.00, 1.67)	0.048
		N	Mea (95% CI)			Difference (95% CI)	p	N	Mea (95% CI))			Difference (95% CI)	p
Anti-rotavirus IgA GMT (U/mL)	Non-WASH	457	14.8 (13.1, 16.7)			0 (ref)		394	15.6 (13.6, 17.9)			0 (ref)	
	WASH	327	18.5 (15.7, 21.9)			1.81 (1.00, 3.28)	0.050	261	20.4 (16.9, 24.8)			2.03 (1.08, 3.81)	0.027

[11] Baseline seropositivity defined as a pre-vaccine IgA GMT ≥ 20 U/mL.

Ref = reference value

Supplementary figures

Figure S1: Detailed CONSORT flow diagram illustrating selection of infants in rotavirus IgA sub-study from overall population of infants enrolled in the SHINE trial.

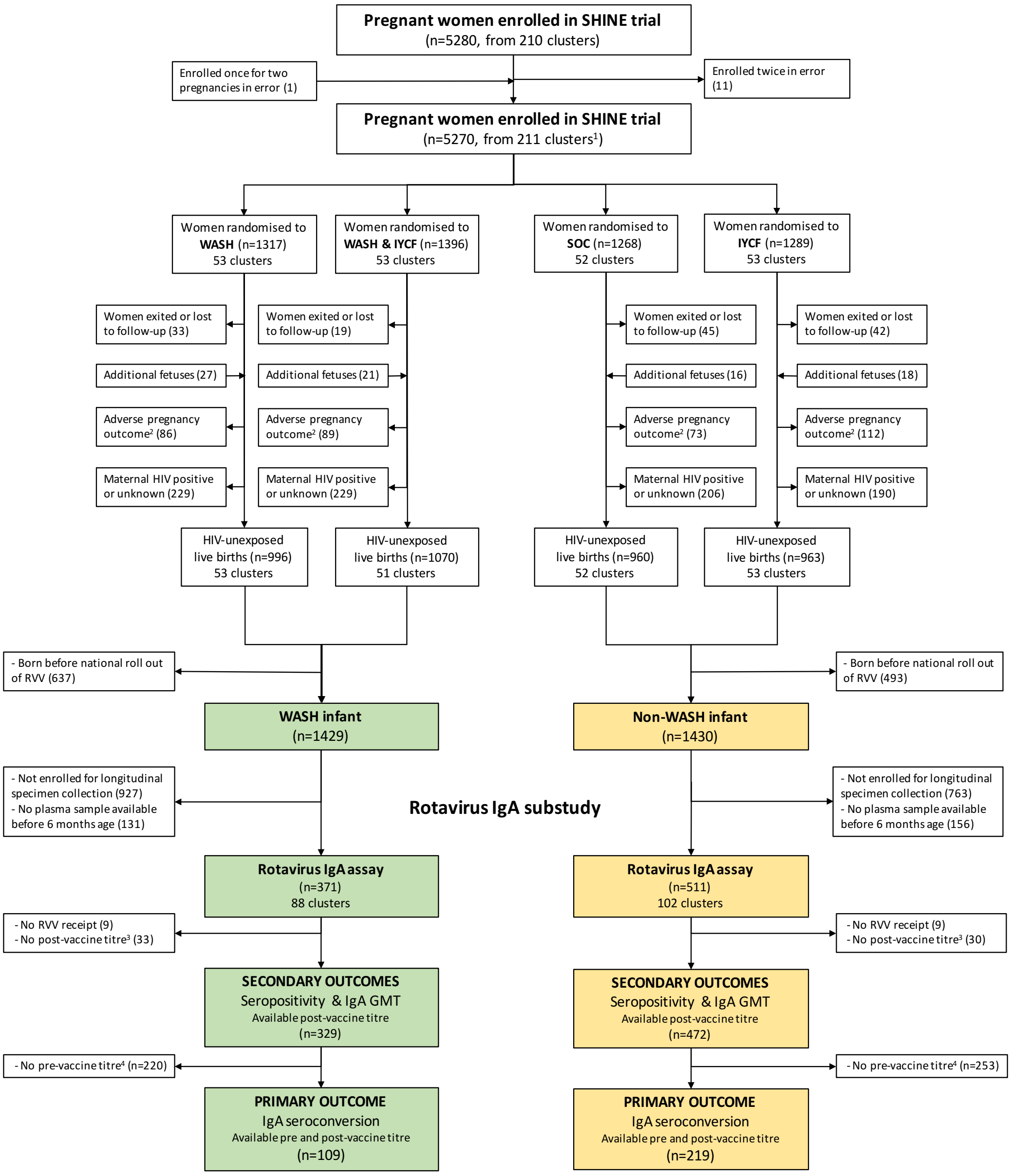

[1] 212 clusters were randomized, 53 in each of the four trial arms. After randomization, one cluster was excluded as it was determined to be in an urban area, one cluster was excluded as the VHW covering it mainly had clients outside the study area, and one more was merged into a neighbouring cluster based on subsequent data on VHW coverage. Three new cluster designations were created due to anomalies in the original mapping. For two of these, the trial arm was clear; the third contained areas that were in two trial arms, and was assigned to the underrepresented arm, resulting in 53 clusters per arm. All of this occurred before enrolment began. When enrolment was completed, however, there was one cluster (SOC) in which no women were enrolled, leaving a total of 211 clusters available for analysis.
[2] Miscarriage, stillbirth or maternal death
[3] No sample available, insufficient post-vaccine sample or assay failure
[4] Insufficient or no pre-vaccine sample available

WASH = Water, sanitation & hygiene; IYCF = infant & young child feeding; SOC = standard of care; RVV = rotavirus vaccine; EED = environmental enteric dysfunction

Figure S2: Anti-rotavirus IgA titres (U/ml) among 328 infants who had both a pre- and post-vaccine plasma sample available

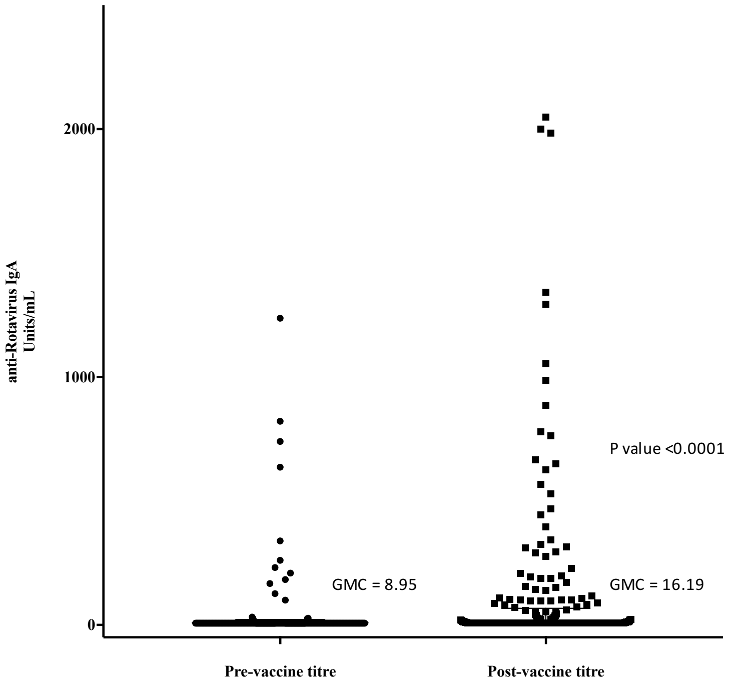

Supplement: ciz140_suppl_Supplementary_Material [file ciz140_suppl_supplementary_material.pdf]
